# Prevalence of anemia among adults at Hawassa University referral hospital, Southern Ethiopia

**DOI:** 10.1186/s12878-018-0133-0

**Published:** 2019-01-08

**Authors:** Misganaw Birhaneselassie Mengesha, Gezahegn Bekele Dadi

**Affiliations:** 1 Department of Medical Laboratory Sciences, Hawassa University College of Medicine and Health Sciences, Hawassa, Ethiopia; 2 School of Nursing and Midwifery, Hawassa University College of Medicine and Health Sciences, Hawassa, Ethiopia

**Keywords:** Prevalence, Anemia, Adults, Ethiopia

## Abstract

**Introduction:**

Anemia is a public health problem in Ethiopia. In spite of the fact that anemia is a common health burden with much severe consequences, the prevalence of the different types of anemia and its severity have not yet been well documented in different parts of the country. The study aimed to assess the prevalence of different types of anemia, including severity and association with age and sex of study population.

**Materials and methods:**

Four hundred anemic patients who are men and non-pregnant women above 15 years of age were selected from patients visiting the laboratory for Complete Blood Count (CBC) investigation. The type and severity of anemia were assessed based on red cell indices and haemoglobin levels respectively. Data was analyzed using SPSS version 19. Chi square was used at 95% confidence interval, considering *P* < 0.05 statistically significant for association among categorical variables.

**Result:**

The overall prevalence of anemia in the study was 13%. Majority of cases had mild anemia 58.5%, while 19.0%, and 22.5% of the patients had moderate and severe anemia respectively. Overall, the prevalence of mild anemia increases with age, while the prevalence of moderate and severe anemia decreases as age increases. In the present study, the most common anemia was normocytic, which mostly occur in the elderly (61–85) years of age.

**Conclusion:**

The CBC parameters help to diagnose and classify anemia in to major components, which might help for a better treatment practice in developing countries, where additional investigations are not available for a reliable diagnosis and classification of anemia. Despite resource limitations in developing countries, additional anaemia work up such as iron studies and markers of inflammation, will provide a more efficient diagnosis of anaemia.

## Introduction

Globally, anemia affects more than 2 billion people accounting for over a quarter of the world population [1, 2]. The highest prevalence of anemia exists in the developing world where its causes are multi-factorial. Anemia is responsible for significant morbidity and mortality, particularly in less developed countries. Anemia causes many complications and has been related to reduced work capacity, reduced ability to execute activities of daily living, reduced cognitive function and fatigue among others [3, 4].

In Africa, prevalence of anemia is diverse. A study in Uganda reported a prevalence of 16.8 to 33.8% anemia in adult men and women [5]. Another study also reported anemia in older people, 12.5% in males and 13.2% in females in South Africa [6] and 23% prevalence in Zimbabwe general population [7]. A study from Ghana reported a 53.2% prevalence of anemia in non-pregnant women [8]. Similar studies have been reported in Ethiopia, 17 to 52.3% prevalence of anemia [9–11]. In addition, data from WHO indicated prevalence of anemia among non-pregnant women in Ethiopia was 23.3% as of 2016. Its highest value over the past 26 years was 47.50% in 1990, while its lowest value was 21.40% in 2012 [12].

Despite the high prevalence and serious consequences of anemia, there have been few reported studies assessing the effectiveness of anemia prevention and control programs in developing countries. Prevention and control of anemia is essential to reduce its consequences which further require effective treatment, and management of patients. Likewise, effective diagnosis of anemia is the main avenue for proper treatment and management of anemic patients [13, 14].

Even though anemia places a significant burden in many developing nations with much severe consequences, the magnitude of the different types of anemia and its severity have not yet been well documented in different parts of Ethiopia [11, 15]. Therefore, more work ups on anemia are required to produce national data. There is a need to define the current magnitude of anaemia and aim for its reduction. Tackling this problem from the point of its diagnosis and classification for better treatment and management of patients with anemia would be essential.

The works assess the current magnitude of the problems, its severity and categories for ease of diagnosis and monitoring of anemia among clinicians. This data will be required for health planning and preparation of local guidelines or algorithms for diagnosis. It draws knowledge to consider the implications for treatment guidance, and represents epidemiological data on the prevalence of anemia and its different forms. The present study therefore, aims to study the occurrence of different types of anemia in this particular study population.

## Materials and methods

### Description of the study

The study is intended to assess the prevalence of different types of anemia, including severity and association with age and sex of study participants, and other CBC parameters as variables. This study used data from Complete Blood Count (CBC) performed by hematology analyzer (Cell Dyn 1800) to determine the prevalence of different types of anemia treated at the Hawassa University Referral hospital outpatient department (OPD). All the procedures were performed following standard operating procedures and protocol for hematology analyzer.

### Study subjects

The study subjects were patients who visited the laboratory for CBC investigation, of whom anemic patients were selected to calculate the prevalence and for further investigation on anemia. Those study subjects were sourced from patients visiting the OPDs of the Referral hospital for various clinical examinations. The OPD laboratory of the Referral hospital performs about 30–40 CBC investigations a day on average. During the data collection period (2016, March/April/May) 3076 CBC were performed, out of which 400 anemic subjects were selected for further analysis and study.

#### Study design

Study type: Cross sectional laboratory based study was undertaken on patients visiting the laboratory for CBC investigation. Sampling procedure: Convenient sampling was used. Every patient with CBC result was considered daily. Participants were recruited consecutively. Then, patients with anemia were selected and prevalence was calculated from the total patients with CBC during the study period. Sample size: Using the prevalence formula, z^2^pq/d^2^ and a maximum p of 0.5, the maximum sample size for a cross sectional survey is about 384 at 95% CI, where normal standard deviation is 1.96 and degree of freedom of 5%. The study however considered a contingency of 5% which makes the total sample size required about 400.

### Inclusion criteria

Any patient with anemic result based on the HGB would be involved in the study. Adult men and women who were more than 15 years of age were included in the study. Infants and young children including pregnant women have variable CBC and other hematological values due to physiological and other conditions *and so* were excluded. Hence, it was preferred to include adult male and female who are non –pregnant.

### Procedure

Experienced laboratory personnel collected 5 ml of blood sample for CBC. Anemic patients based on HGB result were selected for further analysis of the CBC values. The CBC reports were registered [Hemoglobin (HGB), Hematocrit (HCT), Mean Cell Volume (MCV), Mean Cell Hemoglobin (MCH), Mean Cell Hemoglobin Concentration (MCHC), Red Cell Distribution width (RDW), Red Blood Cell count (RBC count), White Blood Cell (WBC count), Platelet (PLT) and Differential Leukocyte Count] for each patient. The first step in approaching anemia is to classify the process as microcytic (MCV, < 80 fL), normocytic (MCV, 80–100 fL), or macrocytic (MCV, > 100 fL), which this exercise markedly narrows the differential diagnosis that needs to be considered in each patient [16–18].

Anemia was defined in accordance with World Health Organization (WHO) criteria [19] as a hemoglobin concentration less than 12.0 g/dL in women and less than 13.0 g/dL in men. Women who had HGB value between 11 and 12 g/ dL, 8–11 g/dL, and < 8 g/dL were categorized as having mild, moderate, and severe anemia, respectively. For men anemia < 13.0 g/dl, mild 11–12 g/dl, moderate 8–11 g/dL, and severe < 8 g/dl was considered to classify the severity of anemia in this study [20–22].

### Ethical consideration

Ethical clearance was obtained from IRB (Institute Review Board) of Hawassa University College of Medicine and Health Sciences. Patient consent was obtained and additional support was sought from the laboratory management of Hawassa University Referral hospital.

### Data analysis

Data was collected in a format prepared for CBC results, age and sex of patients. Data was entered in to Excel for cleaning, and was analyzed using SPSS version 19. Chi square was used to see statistical association among variables. Statistically significant association among categorical variables was observed at 95% CI, *p* = 0.05 level of significance.

## Result

**Fig. 1:**
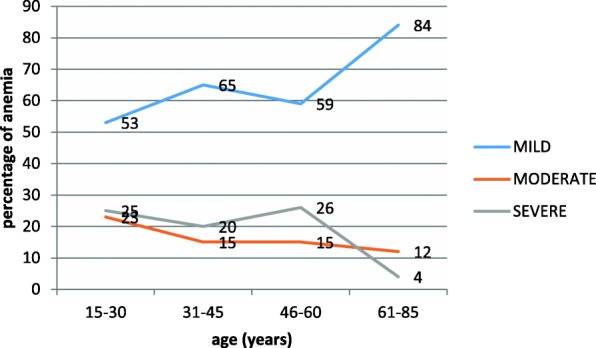
Prevalence and severity of anemia by age

A total of 400 anemic patients were considered in the study. Amongst, 58% were male, and majority were in 15–30 years of age group. The overall prevalence of anemia in the study area was 13%. This study reported, majority of cases had mild anemia 58.5%, while 19.0 and 22.5% of the patients had moderate and severe anemia respectively. Mild anemia is the highest in both males and females. Moderate anemia is much more common in females than in males. The prevalence of severe anemia, however, is slightly higher in males than in females [Table 1]. The prevalence of mild anemia in the study population is much higher than the prevalence of moderate and severe anemia, and even than the combination of the two (moderate – severe). Overall, the prevalence of mild anemia increases with age, while the prevalence of moderate and severe anemia decreases as age increases (Fig. 1).

**Table 1:** Prevalence of mild, moderate and severe anemia by study subject characteristics with *P* value

Study subjects		N (%)	Degree of anemia			*p*-value	95% CI
			Mild N (%)	Moderate N (%)	Severe N (%)		
Age	15–30	215 (54%)	113(52.6)	49 (22.8)	53 (24.7)	0.038	0.000–0.012
	31–45	95(24%)	62(65.3)	14 (14.7)	19 (20.0)		
	46–60	65(16%)	38(58.5)	10 (15.4)	17 (26.2)		
	61–85	25(6%)	21(84.0)	3 (12.0)	1(4.0)		
Gender	Female	168 (42%)	89 (53.0)	42(25.0)	37 (22.0)	0.030	0.013–0.047
	Male	232 (58%)	145 (62.5)	34 (14.7)	53 (22.8)		
MCV	< 80.0	109 (27%)	49 (45.0)	32 (29.4)	28(25.7)	0.001	0.260–0.350
	80.0–100.0	248(62%)	170(68.5)	39 (15.7)	39 (15.7)		
	> 100.0	43 (11%)	15 (34.9)	5 (11.6)	23 (53.5)		
MCH	< 26.0	138 (35%)	67 (48.6)	39 (28.3)	32 (23.2)	0.001	0.222–0.308
	26.0–32.0	208 (52%)	145 (69.7)	28 (13.5)	35 (16.8)		
	> 32.0	54 (14%)	22 (40.7)	9 (16.7)	23 (42.6)		
MCHC	< 31.0	184 (46%)	96 (52.2)	44 (23.9)	44 (23.9)	0.004	0.170–0.250
	31.0–36.0	206 (52%)	136 (66.0)	29 (14.1)	41 (19.9)		
	> 36.0	10 (3%)	2 (20.0)	3 (30.0)	5 (50.0)		
RDW	11.0–14.0	74 (19%)	61 (82.4)	7 (9.5)	6 (8.1)	0.001	0.000–0.007
	> 14.0	326 (82%)	173 (53.1)	69 (21.2)	84 (25.8)		
WBC	5.0	96 (24%)	40 (41.7)	135 (67.2)	59 (57.3)	0.001	0.000–0.012
	5.0–10.0	201 (50%)	19 (19.8)	34 (16.9)	23 (22.3)		
	> 10.0	103 (26%)	37 (38.5)	32 (15.9)	21 (20.4)		

Note: All participants were aged 16 or older. Age 15 was presented to make a clear demarcation in age groups with 15 interval

Based on the MCV, majority of the anemia 62% were normocytic while 27 and 11% of them were microcytic and macrocytic respectively. Whereas, the MCH and MCHC values respectively showed 35 and 46% of the patients had hypochromic anemia. However, 52% of the anemic subjects had normochromic anemia, equally based on both MCH and MCHC values, which is the majority (Table 1). According to the RDW, 81% of the anemic cases demonstrated an RDW of > 14.0 which reflects the red cells in those patients showed anisocytosis. In addition, 26% of the anemic patients had a high WBC count (> 10,000/mm3), while 24% had low WBC count (Table 1).

As depicted in Table-1, most of the anemic subjects showed a normal MCV which reported the most common anemia in the study subjects was normocytic. While the same large group of anemic subjects demonstrated a normal MCH (52%) and MCHC value (52%) which similarly reported a normochromic anemia. Accordingly, this study indicated the leading anemia type among the study population was normocytic normochromic. Besides, this study showed 27% of the study subjects had microcytic anemia (MCV < 80fl), also 35% and 46% of the study subjects had lower MCH and MCHC values which further demonstrated the second most common type of anemia in the study population was hypochromic microcytic. On the other hand, the macrocytic anemia took a lesser proportion among the study group. It showed (11%) of the study subjects reported an MCV value of more than 100fl (Table 1).

In mild anemia majority (68.5%) of the cells are normocytic. In moderate anemia, majority of the cells are microcytic (29.4%). In severe anemia, most of the cells are macrocytic 54% [*p*=0.001]. Similarly, in mild anemia majority of the cells (70%) are normochromic. In moderate anemia most 28% of the cells are hypochromic [*p*=0.001]. RDW is majorly normal in mild anemia (82.4%), [*p*=0.001] (Table 1). The RDW demonstrated highest when the red cells are microcytic (RDW=96.3%) and macrocytic (RDW=88.4%). Similarly, the RDW showed highest result when the red cells are hypochromic (RDW=98%), (Table 1).

**Fig. 2:**
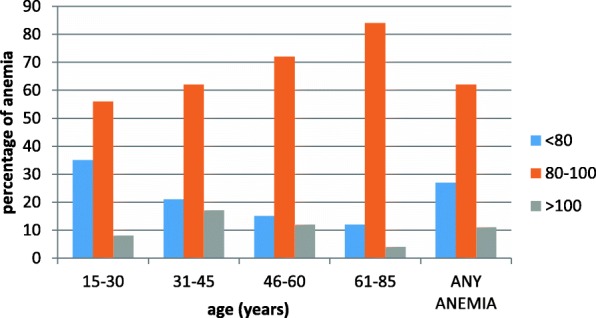
Prevalence of normocytic, normochromic and macrocytic anemia by age

In the present study, the most common anemia was normocytic, which mostly occur in the elderly (61-85) years of age. Microcytic and macrocytic anemias occur more often in younger age group of subjects. In any age group the prevalence of normocytic anemia is higher than microcytic and macrocytic. Prevalence of normocytic anemia increases as age increases. While the second most prevalent anemia microcytic anemia decreases as age increases [*p*=0.001], (Fig 2).

## Discussion

This study registered an overall anemia prevalence of 13 % in the study population. This is considered a minor public health problem according to WHO/CDC classification [23]. This prevalence is much lower than the 30.4% found in the study of Haider performed in nine regional states of Ethiopia in 2010 [11]. Studies in South Africa reported a comparable result 12.6% [24] and 17.5% [25]. A study in Malawi reported a prevalence of anemia in the study population was 16.2% [20]. Adewoyin also reported a 27.3% of anemia in a University Teaching hospital from Nigeria [26]. Prevalence of anemia infact depends on many features such as age, gender, pregnancy, and other related factors. The sampling method used in this study might also have contributed to a lower prevalence of anemia, where the study population included in the study were not specifically diagnosed for having anemia clinically, however, any patient with CBC for any clinical investigation were included in the survey. This is similar with the study from Adewoyin which reported a 12% prevalence of anemia in out-patients. It was further noted that the prevalence of anemia among hospitalized patients is found to be significantly higher compared to out- patients [26, 27] that another factor for the stated prevalence of anemia in the present study could be the subjects were from out-patient departments.

As indicated in the present study the leading anemia was normocytic normochromic. Similar studies reported a high proportion of normocytic anemia; a study in Italy and Brazil reported 88% and 72.3% cases of normocytic anemia [27, 28], another study also reported a 46% prevalence of normocytic normochromic anemia as the most common type of anemia [29]. On the contrary, other studies reported the vast majority of anemia cases were microcytic, which is the second most prevalent anemia in this study [22, 30]. The present study also showed 11% of macrocytic anemia. In comparison a study reported 1.7% to 3.6% macrocytic anemia [31], this study showed there is a higher proportion of macrocytic anemia in the study population than in other studies which needs to be further investigated to identify a possible reason of macrocytic anemia in the study area. Normochromic normocytic anemia dominate through any age group, hypochromic microcytic and macrocytic anemias occur more often in younger age group of subjects, [*p*=0.001]. Studies have shown microcytic and hypochromic anemia more often associated with iron deficiency anemia, but there can be more likely causes for it [32]. Likewise, from the survey based on the higher level of microcytic anemia in the younger patients compared to older patients, it is assumed that IDA is more common in younger patients.

Mild anemia is the most common type in the present study, and severe anemia is more common than moderate anemia. This finding was similar with a study from Nigeria which reported most cases of anemia observed were mild in severity and that 14.9 % of all anemic patients had severe form of anemia [26]. Among the age groups, most of the anemia (54%) occurs in the 15-30 years group and the least (6%) prevalent anemia is found in the elderly who are 61-85 years old patients. Other studies have reported 11.8%, 5.4% and 8 to 44% prevalence of anemia in the elderly which is comparable with our finding [27, 33, 34]. The present study showed mild anemia increases with age and mild anemia affects one out of sixteen elderly individuals.

Similar figures have been reported in other studies [35, 36]. Anemia in the elderly might be considered as a normal consequence of aging, and treatment might be ignored for the true cause of anemia. Other studies have indicated ACD (anemia of chronic disease) is the most common form of anemia in the elderly [37, 38]. Besides, as mild anemia is the most frequent anemia in this study the possible causes underneath need to be investigated including the young age group. Moderate and severe anemia constitutes 40% of the anemia in the survey. These forms of anemia are common in the 15-30 young age group, which affects the productive age group in the society. These group of anemic patients should get attention as they affect patients much more than the mild form of anemia. A great proportion of the moderate and severe anemia belong to the microcytic and macrocytic form of anemia.

In addition, most of severe anemia associated with macrocytic anemia, vise versa; in macrocytic

anemia most of the cases of anemia are severe. In this study severe anemia was found to be more prevalent than moderate anemia. The study from Nigeria however reported moderate anemia is more common than severe anemia unlike this study [26]. Another study reported severe anemia was rare, there being only 22 cases for a prevalence of 0.3% [27]. A study in southern Ethiopia also reported there was no severe anemia among pregnant women [39]. This study however, reported a higher proportion of macrocytic anemia in relation to other studies which should get an attention for its possible causes. The occurrence of a higher proportion of macrocytosis can be due to presence of spherocytosis, due to folate and vitamin B12 deficiency and other reasons. Moreover, this study elucidated macrocytic anemia is largely associated with leukopenia. In leukopenic patients 25% of the cells are macrocytic, unlike 6% in leukocytosis and normal white blood cell patients [*p*=0.004]. This additionally suggests the possibility that this group of patients might have vitamin B12/Folate deficiency as significant leukopenia is a laboratory feature in megaloblastic anemia.

The RDW value was very high throughout the sample measurements that (82%) of the samples

demonstrated a higher than normal RDW (>14.0). The highest RDW was registered in hypochromic anemias 96%, and 88% of macrocytic anemia, than in normocytic anemia 74%, [*P*=0.001]. This study demonstrated the prevalence and severity of anemia is associated with RDW measurement, and severe anemia is associated with a higher degree of anisocytosis. Another study also suggested, MCV should be used together with the RDW, thus directing the interpretation of the variation in the size of red blood cells and hence anemias [40, 41].

Roughly, the CBC report helped in classification of anemia in to three major components. The study also showed these entities of anemia occur in the OPDs of the Referral hospital from the surrounding community. The CBC is not quite suffice to properly identify and reach to a more accurate and precise diagnosis of anemias, but it is still useful to use the CBC for diagnosis and classification of anemia. Additional investigations remain required for proper diagnosis, treatment and management of anemia in developing countries. The iron-panel tests such as Ferritin, Vitamin B12, Folate and the like are fundamental investigations to be carried out for prompt diagnosis and classification of anemia.

## Conclusion

The prevalence of anemia in the study group was considerable. The study identified different types of anemia in good prevalence in the area. The CBC help to diagnose and classify anemia in to major components, which help for a better treatment practice in developing countries, where resources are limited. However, it does not able to diagnose and confirm other entities of anemia, and hence additional investigation methods are essential for efficient diagnosis and classification of anemia, and thus for better management and monitoring of anemic patients.

## Abbreviations

CBC: Complete Blood Count
CDC: Center for Disease Control
HCT: Hematocrit
HGB: Hemoglobin
IRB: Institute Review Board
MCH: Mean Cell Hemoglobin
MCHC: Mean Cell Hemoglobin Concentration
MCV: Mean Cell Volume
OPD: Outpatient Department
PLT: Platelet
RBC count: Red Blood Cell count
RDW: Red Cell Distribution Width
WBC count: White Blood Cell
WHO: World Health Organization
